# Exploiting lipopolysaccharide-induced deformation of lipid bilayers to modify membrane composition and generate two-dimensional geometric membrane array patterns

**DOI:** 10.1038/srep10331

**Published:** 2015-05-27

**Authors:** Peter G. Adams, Kirstie L. Swingle, Walter F. Paxton, John J. Nogan, Loreen R. Stromberg, Millicent A. Firestone, Harshini Mukundan, Gabriel A. Montaño

**Affiliations:** 1 Center for Integrated Nanotechnologies, Los Alamos National Laboratory, Los Alamos, NM, 87545, USA; 2 Department of Biology, University of New Mexico, Albuquerque, NM 87131, USA; 3 Center for Integrated Nanotechnologies, Sandia National Laboratories, Albuquerque, NM, 87185, USA; 4 Center for Biomedical Engineering, University of New Mexico, Albuquerque, NM 87131, USA; 5 Physical Chemistry and Applied Spectroscopy, Los Alamos National Laboratory, Los Alamos, NM, 87545, USA; 6 New Mexico Consortium, Los Alamos, NM, 87545, USA

## Abstract

Supported lipid bilayers have proven effective as model membranes for investigating biophysical processes and in development of sensor and array technologies. The ability to modify lipid bilayers after their formation and *in situ* could greatly advance membrane technologies, but is difficult via current state-of-the-art technologies. Here we demonstrate a novel method that allows the controlled post-formation processing and modification of complex supported lipid bilayer arrangements, under aqueous conditions. We exploit the destabilization effect of lipopolysaccharide, an amphiphilic biomolecule, interacting with lipid bilayers to generate voids that can be backfilled to introduce desired membrane components. We further demonstrate that when used in combination with a single, traditional soft lithography process, it is possible to generate hierarchically-organized membrane domains and microscale 2-D array patterns of domains. Significantly, this technique can be used to repeatedly modify membranes allowing iterative control over membrane composition. This approach expands our toolkit for functional membrane design, with potential applications for enhanced materials templating, biosensing and investigating lipid-membrane processes.

Supported lipid bilayers (SLBs) are simple model membranes^1^,^2^ with lateral lipid mobility^3^ that allow investigation of biologically-relevant membrane processes using surface-based techniques. For example, topographical mapping of SLBs by atomic force microscopy (AFM)^4^,^5^,^6^ and fluorescence microscopy of SLBs doped with dyes^7^ has been used to reveal domain formation within membranes. Compartmentalization of SLBs into diffusion-limited array patterns allows for spatial segregation of membrane function, which has proven useful for many applications including biosensors and directing cell adhesion^8^,^9^,^10^. Several techniques have been developed for patterning SLBs, each with their own advantages and challenges. Physical barriers to compartmentalize membranes can be created on substrates prior to SLB formation, e.g., lithographically etched^11^, deposited metals and printed protein barriers^12^. Alternatively, after formation of SLBs, defects can be selectively introduced to confine membrane regions, e.g., UV exposure through a patterned mask^12^,^13^, focused lasers^14^ or scanning probe lithography^15^. Another possibility is the direct deposition of lipid bilayers onto a substrate as microscale arrays by microcontact printing^12^,^16^,^17^,^18^ or as nanoscale patterns using advanced techniques such as dip-pen nanolithography^19^. Whichever technique is used to pattern lipid bilayers, any ‘empty’ regions can be subsequently ‘backfilled’ with secondary components to produce multi-component SLBs^12^,^13^,^15^,^18^.

Each membrane patterning technique mentioned above affords unique opportunities in SLB studies ranging from investigating fundamental biophysics to applied sensor technology. However, it is challenging to modulate the membrane composition after initial patterning. Since the techniques described above typically rely upon, for example, initial boundary constraints to generate patterns or post-formation mechanical disruption of the SLB, continual modulation of the membrane composition can be difficult and/or time- and labor-intensive. In addition, building secondary patterns in spatial proximity to the first is challenging due to sample registry. Fodor *et al*.^20^ demonstrated a spatially-addressable array of 1024 different chemical products on a solid support by multiple cycles of photolithography, however this required the use of many different photomasks, precise sample alignment and complex chemistry (and is not amenable to aqueous samples, e.g. biomembranes). Whilst theoretically possible using similar approaches, *in situ* modification (i.e., under aqueous conditions) of SLB composition and hierarchical patterns of membranes containing confined regions of different compositions have not previously been demonstrated. This may be due to the difficulty of repeated modification of soft biological membrane materials, which are easily damaged by processes involving drying or solvents. In this work, we have developed a new process that overcomes many of these challenges.

Lipid membranes can be disrupted by various nanoparticles^21^, including membrane-inserting biological amphiphiles such as pore-forming toxins^22^. We recently showed that lipopolysaccharide (LPS), an important human endotoxin^23^,^24^, inserts into SLBs and causes disruption of the membrane leading to the formation of micron-sized holes. Note that the fluidity and continuity of the remainder of the membrane is maintained^25^. We conceived of the idea to utilize the inherent ‘self-aggregation’ of LPS in SLBs followed by membrane destabilization as a means to controllably modify SLBs, post-formation. In designing this study, we surmised that using LPS as a tool to modify membranes could offer several advantages as a ‘gentle’, aqueous-based modification of lipid bilayers.

Herein, we report the use of this biological amphiphile to selectively modify and pattern membranes in a unique and previously unexplored manner. This report serves as a proof-of-concept demonstration that biological molecules can be exploited in a novel manner for *in situ* modification of biomembrane materials.

## Results

**Figure 1:**
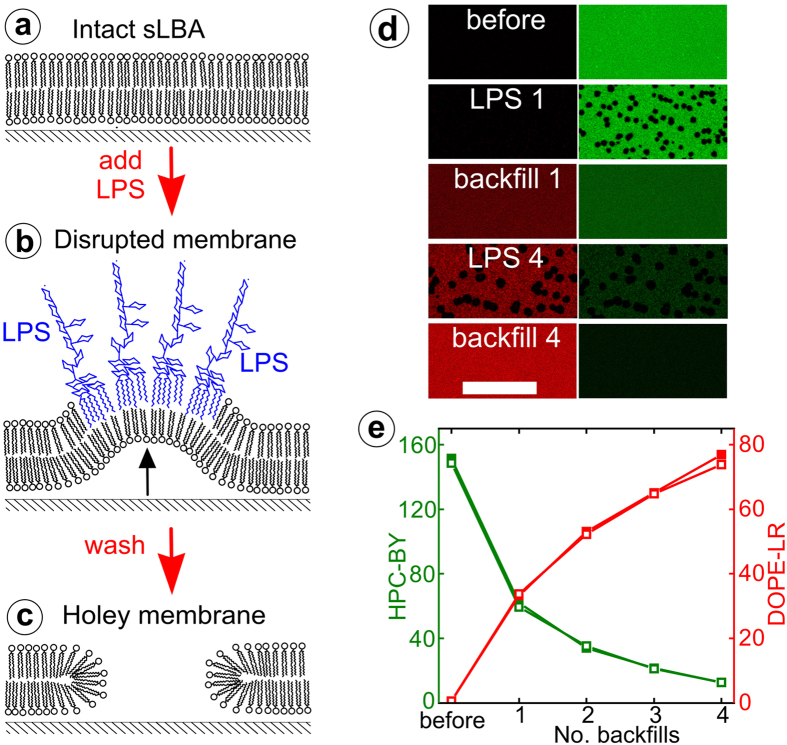
Lipopolysaccharide-induced void formation and backfilling. Schematic of the void formation process induced by LPS (**a-c**) and LSCM data showing backfilling with fluid-phase lipids (**d-e**). A DOPC SLB is planar and continuous (**a**). After LPS addition, it inserts into the lipid bilayer and clusters, inducing membrane curvature (**b**). After washing the surface with buffer, voids are formed from the curved regions (**c**). Using DOPC doped with fluorescent dyes, voids are observed by LSCM. Multiple cycles of void formation and backfilling can be used to alter the membrane composition, as shown in (**d**) by almost complete exchange of the lipid dye incorporated in the SLB. Here, a DOPC SLB doped with 0.5% (mol/mol) 2-BODIPY-1-hexadecanoyl-*sn*-glycero-3-phosphocholine (HPC-BY) (*green*) was treated with 500 μg/mL LPS, washed, and then backfilled with DOPC liposomes doped with 1% (mol/mol) 1,2-dioleoyl-*sn*-glycero-3-phosphoethanolamine lissamine rhodamine (DOPE-LR) (*red*) and washed again. Incremental loss of HPC-BY lipid dye and introduction of DOPE-LR lipid dye was observed by acquiring images after each LPS treatment (*“LPS #”*) and then each subsequent backfill (*“backfill #”*). In (**d**), separate image channels show either DOPE-LR (*left column*) or HPC-BY (*right column*), displayed to an equal colour-scale (x-y scale bar: 50 μm). Analysis of the relative fluorescence intensity after each backfill stage is shown graphically in (**e**). For each data point, the mean intensity from 5 different fields across the sample is averaged. Standard deviations calculated were smaller than the data points (0.4 – 3.8). Two independent experiments are shown (*solid* and *open* data points).

Firstly, a fluid-phase SLB is formed on a hydrophilic glass substrate from the phospholipid 1,2-dioleoyl-*sn*-glycero-3-phosphocholine (DOPC) using the vesicle spreading method (Fig. 1a). Treatment of the SLB with LPS leads to its insertion into the membrane, clustering and localized membrane de-stabilization (Fig. 1b). Upon sample rinsing, micron-sized voids in the membrane are formed (Fig. 1c), as previously reported^25^. Samples were characterized using laser scanning confocal microscopy (LSCM) for large area fluorescence imaging at moderate (~250 nm) resolution and AFM for topographical imaging at nanometer-resolution. Voids were observed in our experiments as areas lacking fluorescence in LSCM images and pits within the otherwise continuous surface in AFM topographs (Fig. 1d and Supplementary Fig. S1)^25^. We demonstrated that these LPS-generated voids could be backfilled with various components, analogous to other membrane patterning studies^12^,^13^,^15^,^18^, which allowed the generation of static domains of proteins or gel-phase lipids, or alternatively ‘healing’ the membrane using fluid-phase lipids (Supplementary Fig. S2). One advantage of our method is that multiple void creation and backfilling steps may be performed in an iterative manner resulting in dramatic modification of the membrane composition. Such modification was demonstrated by changing the bulk membrane dye composition over multiple cycles of void formation and backfilling with fluid-phase lipids (Fig. 1d-e). Over the course of four cycles the intensity of the green lipid dye is decreased from ~150 to ~10 counts, whilst the red lipid dye increases from zero to ~75 counts (measured as relative intensities from LSCM). Thus, our method represents a novel method to dramatically adjust the bulk lipid content of an SLB, with potential implications for creating, for example, regenerating membrane biosensors.

**Figure 2:**
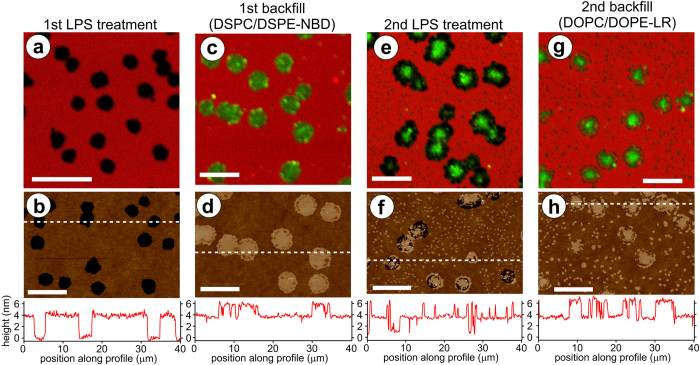
Hierarchical lipid domain assembly by LPS lithography and DSPC backfilling. LSCM and AFM images of representative areas at different stages of the lithography/backfilling process. Here, the initial SLB was formed from fluid-phase DOPC (phase transition T_m _= –17 °C^29^), doped with 1% DOPE-LR for fluorescence imaging (*red*). Backfilling was performed by adding a solution of DSPC liposomes doped with 1% (mol/mol) 1,2-distearoyl-*sn*-glycero-3-phosphoethanolamine-N-[7-nitro-2-1,3-benzoxadiazol-4-yl] (DSPE-NBD) (*green*) at ~60 °C, which quickly cools to below their phase transition temperature (T_m _= 55 °C^29^), forming gel-phase domains. The lipid dyes chosen here favour different phases: the DOPE-LR labels the fluid phase, whereas the DSPE-NBD labels the gel phase. (**a**)-(**b**) DOPC/DOPE-LR SLB after LPS treatment; (**c**)-(**d**) after backfilling with DSPC/DSPE-NBD liposomes; (**e**)-(**f**) after a second LPS treatment; (**g**)-(**h**) after second backfilling with DOPC/DOPE-LR liposomes. Note, the ‘small islands’ of DPSC of 1 μm size referred to in the text appear as raised domains in the AFM data (f), but in LSCM data (e) the expected DSPE-NBD (green) fluorescence is not apparent due to the relatively weak intensity of NBD emission. (**a**), (**c**), (**e**) and (**g**) are composite dual channel LSCM fluorescence images, adjusted for contrast. (**b**), (**d**), (**f**) and (**h**) are AFM topographs, with the colour scale representing a z-range of 10 nm and with height profiles shown for the *white dashed lines* across sections of the images. Scale bars in all images are 10 μm.

Hierarchical 2-D membrane organizations of controlled compositions are of significant interest for mimicking and studying biological systems, for example, the organized protein domains in the membranes of photosynthetic bacteria^26^,^27^,^28^. In order to accomplish such a task, it is necessary to dictate insertion of multiple desired components into positions adjacent to each other. In our experiments, LPS-treated membranes appeared to demonstrate memory, whereby after voids are apparently healed by backfilling with fluid-phase lipids, a second LPS treatment often produced voids at the same position (rate of recurring holes over 4 LPS treatments was ~70%, see Supplementary Fig. S3). After multiple LPS treatments, holes appeared/disappeared/reappeared but interestingly no ‘new’ holes occurred (holes at different locations), indicating that the first LPS treatment pre-determines hole positioning for subsequent LPS treatments (Fig. S3) This tendency of LPS to act at the same positions after multiple treatments suggested that nanoscale defects may remain and led to the idea that we could direct void formation to the interface between immiscible lipid phases (irregular areas of lipid-lipid mismatch). Indeed, by performing multiple cycles of void formation and backfilling with gel-phase lipids we were able to generate concentric membrane domain arrangements, as described below. Firstly, voids in a fluid-phase DOPC SLB were produced by LPS treatment (Fig. 2a-b) and then lipid microdomains were formed by backfilling with liposomes of gel-phase 1,2-distearoyl-*sn*-glycero-3-phosphocholine (DSPC)^29^ (Fig. 2c-d). Note that by mixing incompatible lipid phases one interrupts normal lipid diffusion, producing a long-lived metastable phase separation (no significant change was observed over the course of an experiment of several hours). A second treatment with LPS targeted the DOPC/DSPC interface, forming a moat-like gap around the gel-phase DSPC domains (Fig. 2e-f). AFM height profiles confirmed the expected thickness of fluid- and gel-phase membranes relative to the substrate (3-4 nm and 5-6 nm, respectively)^4^,^6^. Note that after this second LPS treatment, the existing DSPC domains appeared to shrink and some smaller DSPC islands (~1 μm) appeared (see Fig. 2e-f). A plausible explanation for this unexpected result is that LPS induces movement of some of the DSPC already present in the membrane (at DSPC domain peripheries and/or mixed in with DOPC) to form these smaller phase segregated areas. A second backfilling treatment using DOPC liposomes regenerated the fluid-phase portion of the lipid membrane and led to formation of irregularly shaped gel-phase microdomains (Fig. 2g-h). Time-lapse LSCM of the second backfilling process showed that fluid-phase lipids localized at the perimeter of gel-phase domains followed by outward spreading (Supplementary Fig. S4). Alternatively, a second backfill with gel-phase lipids could be used to enlarge the existing gel phase domains (Supplementary Fig. S5). We could also generate multi-component protein domains with ring-like hierarchy, by multiple backfill cycles with proteins rather than lipids (Supplementary Fig. S6). These results demonstrate the utility of LPS as a means for preferentially generating spatially-arranged membrane defects for SLB design as well as provide an interesting narrative of the biophysical properties of LPS, discussed later. Further, being that this process relies purely on spontaneous physical/chemical interactions of biomolecules, it overcomes the burden of domain registry previously described as necessary to obtain spatially-arranged SLBs.

**Figure 3:**
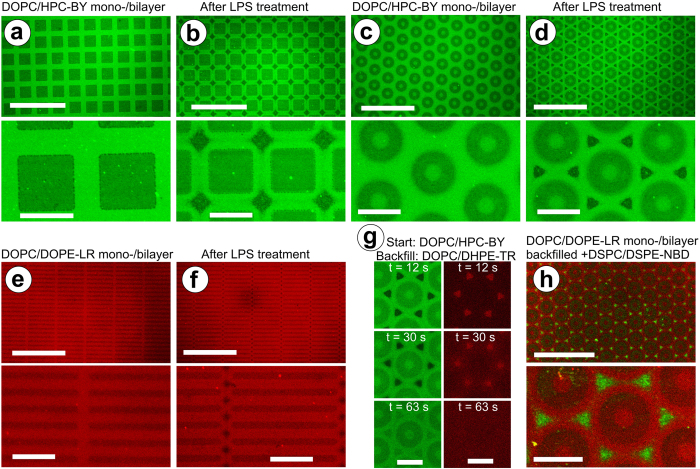
Geometric control over LPS-induced array patterned membranes. LSCM images of different array patterns of DOPC lipid monolayers (*dark*), surrounded by lipid bilayers (*bright*), doped with either 0.5% HPC-BY or 1% DOPE-LR, as labelled. Box pattern (square-packed), before (**a**) and after (**b**) LPS treatment. Washer pattern (hexagonally-packed), before (**c**) and after (**d**) LPS treatment. Bar pattern (rectangular-packed), before (**e**) and after (**f**) LPS treatment. Time-lapse series (**g**) showing ‘repair’ of voids in washer pattern (as in (**d**)) by backfilling with DOPC liposomes doped with 0.5% Texas Red 1,2-dihexadecanoyl-*sn*-glycero-3-phosphoethanolamine (DHPE-TR); separate image channels shown in left/right *columns*. Washer pattern of DOPC/DOPE-LR treated with LPS, after backfilling with DSPC liposomes doped with 1% DSPE-NBD (*DSPC-NBD*), shown as composite dual channel images (**h**). Scale bars are 100 μm (*upper panel*) and 20 μm (*lower panel*) for (**a**)-(**f**) and (**h**) and 20 μm for (**g**).

Generation of array patterns of multi-component membrane systems is highly desirable, but requires controlled, sophisticated patterning^8^,^9^. Control over LPS action was achieved in multiple ways, including incubation time and concentration, revealing trends for the size and number of voids (Supplementary Fig. S7 and ref. 25). For effective patterning of SLBs using LPS, spatial control over void distribution is necessary. Preliminary tests showed that LPS had no effect on supported lipid monolayers (Supplementary Fig. S8). We were able to take advantage of the resistance of lipid monolayers to LPS together with membrane line tension and ordering effects (discussed later) to direct patterning. Traditional soft lithography (micro-contact printing)^16^ was used to pre-pattern the substrate with 2-D arrays of surface chemistry to direct lipid membrane formation^30^ into bilayer and monolayer regions, followed by addition of LPS to enhance the pattern complexity, as follows. A square array pattern of alternating DOPC lipid monolayers (*boxes*) and bilayers (*grid*) was generated (Fig. 3a) by liposome deposition onto a chemically-patterned hydrophilic/hydrophobic substrate^30^. LPS treatment induced the formation of voids only on the defined lipid bilayer grid (lipid monolayer regions were unaffected) (Fig. 3b). Moreover, the voids were diamond-shaped (~8 μm edge length) and located at well-defined positions in the square lattice (Fig. 3b). Void morphology and positioning could be controlled in a top-down manner by the geometric pattern of the lipid bilayer/monolayer. For example, a 2-D hexagonal array of lipids produced triangular voids (~6 μm edge length) after LPS treatment (Fig. 3c-d). A bar pattern of lipids gave rise to diamond-shaped voids of smaller size (~2 μm edge length) than those produced by the more spaced-out square lattice structure (Fig. 3e-f). Backfilling these geometric void patterns resulted in lipid redistributions similar to those described for randomly distributed voids, with the additional advantage that the patterns directed the assembly of further added membrane components. Array-directed introduction of lipids and membrane healing was demonstrated by backfilling with DOPC lipids (Fig. 3g). Arrays of gel-phase domains with long-range 2-D order could be created by backfilling with DSPC (Fig. 3h and Supplementary Fig. S9), transcribing the void pattern into a lipid compositional pattern.

When combined with the described void dependency upon such factors as LPS concentration and incubation time in addition to potential differences with varying lipids and environmental control, the potential for highly tunable ordered arrays of dictated size and shape are possible. Furthermore, as pattern dimensions are increased, one may expect a threshold above which there is no longer any pattern (when large regions of bilayers act as independent SLBs). We did not observe any upper or lower boundary limits at which we no longer observed geometrically-controlled void formation, using the grid patterns reported in the current study (separations ranging from 2-20 μm). Future investigations could study such parameter limits that may indicate thermodynamic or kinetic effects of LPS-membrane interaction. Such experiments will be addressed in the future by our team.

## Discussion

Our results show that LPS-induced membrane deformation can be used to generate complex membrane arrangements within SLBs, post-formation and *in situ*. We demonstrated: (i) change in the bulk lipid composition of an SLB (Fig. 1), (ii) 2-D hierarchical lipid domain arrangements (Fig. 2 and Supplementary Figs. S4-S6), and (iii) microscale 2-D array patterns of lipid domains in defined geometries (Fig. 3 and Supplementary Fig. S9). Below, we discuss the mechanism of action, followed by an assessment of the unique advantages and the potential applications of our technique.

The mechanism for random void formation in SLBs due to LPS insertion, clustering and induction of membrane curvature was recently described^25^; below we discuss how LPS membrane insertion specificity can be used to form controlled, patterned membrane arrangements. *Firstly*, we consider why ring-like arrangements are observed during multiple LPS/backfilling steps (Fig. 2 and Supplementary Figs. S4-S6). LPS, a complex lipoglycan, acts as a gel-phase lipid undergoing microphase separation^4^,^6^, as previously reported^25^,^31^. The saturated fatty acids of each LPS molecule associate with other similar saturated fatty acids resulting in optimal hydrophobic-hydrophobic interactions^25^,^31^, promoting both LPS self-association and its association with DSPC rather than DOPC. We propose that, after insertion and lateral reorganization, LPS molecules cluster at the boundary between the DOPC membrane and the DSPC backfilled domain because this is the most thermodynamically favourable arrangement. This is indirectly observed after rinsing the SLB as an excision of lipids located at the domain periphery, which occurs because LPS clustering induces membrane curvature and instability, as previously proposed^25^. Such a model would be consistent with previous studies that have proposed LPS integration into raft-like domains^32^,^33^ as a means of initiating host immune response. While most models implicate a number of varying cofactors, our results provide further evidence of the tendency of LPS to associate with gel-phase lipidic domains based upon its intrinsic properties in a simple model system. *Secondly*, we consider why LPS has no effect on lipid monolayers (Fig. 3 and Supplementary Fig. S8). A recent study showed that a non-ionic detergent could cause complete destruction of lipid bilayers but had no effect on lipid monolayers, demonstrating the greater stability of monolayers^34^. In a similar manner, we expect that monolayers are resistant to LPS-induced void formation because it is thermodynamically unfavourable to induce curvature in and ‘unzip’ a lipid monolayer because this would expose the hydrophobic lipid tails and alkyl-functionalized substrate to the polar solvent. Importantly, unlike detergent treatment, LPS interaction is shown to allow controlled modification of bilayer areas. *Finally*, we address the LPS-induced generation of voids in well-defined symmetrical arrays as a consequence of the geometry of the patterned lipid bilayers (Fig. 3 and Supplementary Fig. S9). We consider two related phenomena: (i) phase segregation effects, (ii) intrinsic ordering properties of lipid membranes. Microphase separation drives LPS to aggregate in regions that maximize its interactions with itself and minimizes interactions with DOPC lipids, to minimize line-tension (i.e., interfacial tension energy at domains edges) within the membrane. This is consistent with previous reports of LPS leading to enhanced raft formation upon inclusion in lipid mixtures^32^. These regions correspond to positions where the DOPC bilayer is least interrupted by the monolayer domains (large area to circumference ratio), i.e. the geometric centre of patterned regions. A second property that could promote the observed patterns relates to the optimal packing of lipid tails. Prior work has shown that the crystallographic special positions of an organized lipid assembly define regions of maximal short-range apolar disorder of alkyl chain packing^35^. These so-called “chaotic zones” occupy higher symmetry elements of the crystallographic lattice^36^. While a further detailed study is required to fully ascertain the mechanisms involved, such a description is consistent with our data and previous reports.

The simplicity of our new method allows rapid, reproducible membrane modification. Other studies have attempted adjustment of lipid membrane composition, but are often limited to use of certain types of lipids and/or the extent of final membrane modification^37^,^38^. Our described approach significantly advances upon the potential for *in situ* lipid bilayer modification. For pattern-directed adjustments, other techniques could be used in theory to modify membranes post-formation to generate similar arrangements, this is not trivial and has yet to be demonstrated due to practical limitations. For example, multiple cycles of UV-photolithography would require multiple photomask designs and challenging, time-intensive sample re-alignment between stages^20^. Multiple cycles of AFM-lithography^15^ would also be labour-intensive and lack the scalability to produce patterns over large areas. By comparison, the use of LPS as demonstrated in the current study to change the bulk membrane composition or to create randomly-positioned hierarchical lipid domains requires only the exchange of buffers containing LPS and the desired secondary components. If 2-D-ordering is desired, only one additional step is needed (microcontact printing to pre-pattern the substrate). Furthermore, our approach is specific to modification of lipid bilayers, by virtue of using a membrane-inserting amphiphile, whereas other lithography techniques may have undesirable collateral effects, such as alteration of substrate chemistry or damage to other biological components. The advantage of membrane-specificity when using LPS could allow greater flexibility in developing the approach (e.g. combination with other surface functionalities). One may consider whether use of LPS has any residual effects that may affect future products. Our previous study could detect no residual LPS in the membrane after SLB treatment and hole formation^25^ (from a fluorescently-tagged LPS), although quantitative analysis of membrane content using a technique such as mass spectrometry would be required to exclude this possibility. This study focused on one type of fluid-phase lipid and one type of gel-phase lipid, future studies could investigate applicability to a range of lipids.

There are multiple applications that could exploit the advantages of our approach. Use of our technique to locally introduce two or more different membrane components could have applications for studying or mimicking biological processes that require interaction of components in close proximity, for example, enzyme cascade reactions and energy transduction^39^. The ability to repeatedly modify membranes using our technique, particularly at precisely controlled geometric sites, allows the potential for *in situ* adjustments of membrane composition between assays and the re-use of devices built around this platform. For example, SLB-based biosensors^40^ could be regenerated by removal and replenishment of spent components. To scale-up our method, a microfluidic chip^41^ could be designed for optimised flow and exchange of solutions containing LPS and combinations of lipids to allow multiplexed, switchable membrane compositions. This could be applied to running binding assays on massively multi-parallel membrane assemblies.

Fundamental insight into how amphiphiles interact within a membrane was provided, in that a microscale pattern of lipid monolayers and bilayers has long-range order induced by line tension and lipid packing effects sufficient to direct the action of a secondary membrane-inserting amphiphile. The ability to define loci of maximal short-range lipid order coupled with the LPS destabilization affords a novel means to design and create complex patterns possessing variable spacings and feature sizes, *a priori*.

Other biological molecules known to disrupt cell membranes have been investigated using SLBs as model membranes^21^,^22^, however these studies did not probe the parameters controlling the lateral location of membrane disruption. Future studies could investigate the thermodynamic mechanisms of LPS-induced hole formation in detail and determine whether this pattern-directed clustering is a general effect of other amphiphiles or specific to LPS. On a biological level, our results have potential implications on the interactions that may occur between LPS and components in cellular membranes. Our controlled, patterned SLB system could be an effective platform for screening other parameters of potential importance for biophysical interactions of LPS, such as alternative lipid composition^32^, environmental conditions (pH, temperature, ions)^25^, type of LPS (different bacterial species produce LPS with very different structures)^24^ and lipid raft formation^32^.

In summary, LPS-induced membrane deformation has potential applications ranging from membrane biophysics to applied technologies. As a patterning technique, it offers multiple advantages for straightforward, rapid processing resulting in versatile membrane compositions and organization. This new technique exploits localized alterations in alkyl chain packing coupled with destabilization induced by interaction with a secondary membrane amphiphile (here, LPS). The ability to create hierarchically-patterned assemblies of lipids offers significant promise in the generation of robust, adaptable multi-component membranes for biomaterials applications. Fundamentally, these studies clearly show how subtle differences in molecular interactions can be exploited for the creation of complex functional and dynamic membrane materials.

## Methods

### Liposome preparation

All standard and fluorescent lipids were purchased in dry powdered form from Avanti Polar Lipids (Alabaster, AL) or Molecular Probes (Eugene, OR). Fluid-phase liposomes were comprised of DOPC, doped (%mol/mol) with either 0.5% HPC-BY, 0.5% DHPE-TR or 1% DOPE-LR, as stated in the text. Gel-phase liposomes were comprised of DSPC, doped with 1% DSPE-NBD. Liposomes were prepared following established protocols^2^. Briefly, lipids and lipid dyes were dissolved in chloroform, mixed in the desired molar ratios, dried overnight, followed by rehydration in phosphate-buffered saline (PBS, pH 7.4). After three freeze-thaw cycles, the suspension was subjected to probe sonication for 10 min either in an ice bath (for DOPC) or allowed to heat to >60 °C (for DSPC). DOPC liposomes were stored at 4 °C and used within one week; DSPC liposomes were maintained at 60 °C and used within a few hours.

### Substrate preparation

For non-patterned studies, hydrophilic glass coverslips were used as substrates (cleaned with ‘piranha’ solution of 3:1 H_2_SO_4_/30% H_2_O_2_). For patterning, substrates were functionalized by microcontact printing of a hydrophobic silane onto hydrophilic glass coverslips, following established methods^16^, described briefly below. Polydimethylsiloxane stamp pads with different relief patterns were formed by polymerization of dimethylsiloxane on appropriate silicon master patterns, followed by Soxhelet extraction. Stamps were inked with 0.4% (w/v) octadecyltrichlorosilane in toluene and used immediately to print onto hydrophilic glass. Printed substrates were sonicated through a solvent series of toluene, dichloromethane and ethanol and used immediately. Hydrophobic ultrathin ‘imaging spacers’ (Electron Microscopy Sciences, Hatfield, PA) were attached to substrates to create small wells to confine a buffer droplet creating an ‘open’ sample set-up to allow multiple buffer exchanges and top-down access for AFM. SLBs were formed by deposition of 0.5 mg/mL DOPC liposomes onto the substrate for 20 min then washed with PBS.

### Biochemical lithography using LPS and backfilling membrane voids

LPS from *E. coli* serotype O111:B4 (phenol extract), purchased from Sigma-Aldrich (St. Louis, MO), was used to disrupt membranes as previously described^25^. To generate voids in lipid bilayers, DOPC SLBs were incubated with LPS at concentrations of 100-500 μg/ mL in PBS for 20 min and then washed with fresh PBS. Subsequent to characterisation by microscopy, various secondary components were used for backfilling. Globular proteins were used in preliminary backfilling experiments (see Supplementary Fig. S2), by incubating with 0.5 mg/mL bovine serum albumin tagged with an AlexaFluor label (AF594 or AF647) in PBS, followed by washing with fresh PBS. Backfilling with DOPC or DPSC liposomes was by incubation with 0.5 mg/mL liposomes for up to 20 min, followed by washing with PBS. DSPC liposomes were kept at 60 °C until use (above their phase transition temperature)^29^. Adding hot DSPC liposomes to the room temperature DOPC SLB during backfilling allowed DSPC lipids to form a domain contiguous with the surrounding DOPC but complete mixing was prevented as the DSPC cools below its phase transition.

### Fluorescence Microscopy and Atomic Force Microscopy

Samples were maintained in hydrated form, never allowed to dry either during preparation or analysis. LSCM used an FV-1000 inverted optical microscope (Olympus, Tokyo, Japan) equipped with multi-channel photomultiplier detectors, operated in ‘photon-counting mode’ (background noise, 0-2 counts) and a 40 x air objective (NA = 0.95). Excitation was provided by a multi line Ar laser (488 nm, for BY or NBD), a HeNe laser (543 nm, for TR, LR or AF594), or a diode laser (635 nm, for AF647). Appropriate high performance band-pass emission filters were used (505-525 nm for BY and NBD; 560-620 nm for AF594; 655-755 nm for TR, LR and AF647). Fluorescence recovery after photobleaching (FRAP) was performed using manufacturer’s provided software. Quantitative analysis of fluorescence intensity data (Fig. 1d-e) was performed using ImageJ software (open source, v.1.45), as follows. The mean gray value was measured for each image and values from five images acquired from different regions across the sample were averaged for each datapoint and standard deviations calculated. AFM was performed using an MFP-3D-SA system, equipped with a closed loop XY scanner and all-digital ARC2 Controller (Asylum Research, Santa Barbara, CA). All imaging was performed under fluid with probes protruding into the liquid droplet, using Bruker SNL probes (k ~ 0.12 N/m). Parameters were adjusted while scanning to optimize tracking, minimize noise and ensure minimized forces imparted on the membranes. Images were consistent with use of different AFM probes and similar samples leading to robust interpretations of AFM data. Images were processed using the manufacturer’s provided Igor Pro-based software. Graphs were plotted using OriginPro software (v.8.6.0).

## Additional Information

**How to cite this article**: Adams, P. G. *et al*. Exploiting lipopolysaccharide-induced deformation of lipid bilayers to modify membrane composition and generate two-dimensional geometric membrane array patterns. *Sci. Rep*. **5**, 10331; doi: 10.1038/srep10331 (2015).

## Supplementary Material

Supplementary Information
