# Supplementary material for: Exploiting lipopolysaccharide-induced deformation of lipid bilayers to modify membrane composition and generate two-dimensional geometric membrane array patterns
Source: Sci Rep. 2015 May 27;5:10331. doi: 10.1038/srep10331 (PMC4444833; doi:10.1038/srep10331)
Supplement: Supplementary Information [file srep10331-s1.pdf]

Supplementary Information

Exploiting lipopolysaccharide-induced deformation of lipid bilayers to modify membrane composition and generate two-dimensional geometric membrane array patterns

Peter G. Adams[1#], Kirstie L. Swingle[1,2], Walter F. Paxton[3], John J. Nogan[3], Loreen Lamoureux[4], Millicent A. Firestone[1], Harshini Mukundan[5,6] and Gabriel A. Montaño[1]*

[1]Center for Integrated Nanotechnologies, Los Alamos National Laboratory, Los Alamos, NM, 87545;

[2]Department of Biology, University of New Mexico, Albuquerque, NM 87131.

[3]Center for Integrated Nanotechnologies, Sandia National Laboratories, Albuquerque, NM, 87185.

[4]Center for Biomedical Engineering, University of New Mexico, Albuquerque, NM 87131.

[5]Physical Chemistry and Applied Spectroscopy, Los Alamos National Laboratory, Los Alamos, NM, 87545.

[6]New Mexico Consortium, Los Alamos, NM, 87545.

* To whom correspondence should be addressed. E-mail: gbmon@lanl.gov.

# Current address: School of Physics and Astronomy, University of Leeds, Leeds LS2 9JT, United Kingdom

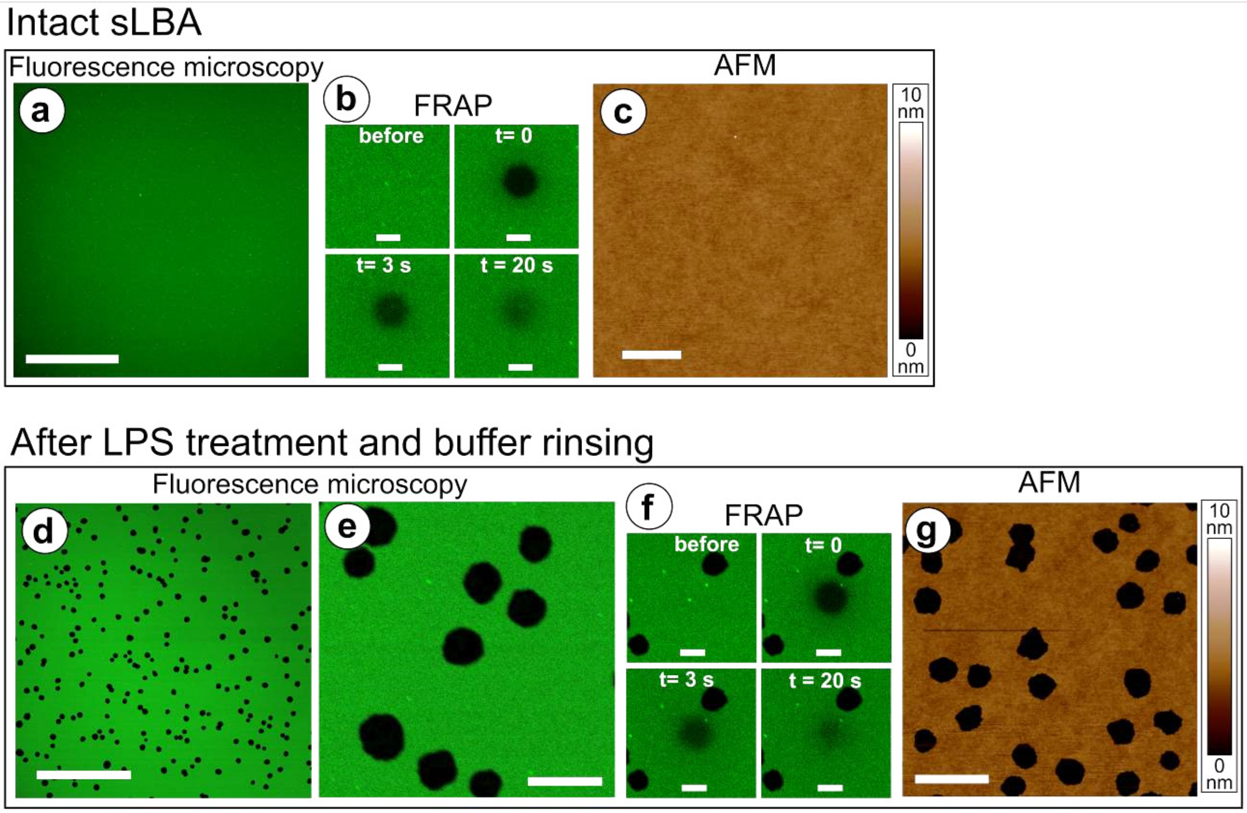

Figure S1. DOPC SLBs (doped with 0.5% HPC-BY, *green*) before and after LPS treatment. (a) LSCM of a DOPC SLB showing homogeneous fluorescence over hundreds of microns. (b) Fluorescence recovery after photobleaching (FRAP) experiments showing high lateral mobility of DOPC within the SLB, indicative of a high quality continuous membrane. FRAP was performed using manufacturer's provided software. (c) AFM topograph of a DOPC SLB showing that it is relatively smooth and defect free over many microns. (d) LSCM after LPS treatment of a DOPC SLB, showing voids of 1-10 µm width observed as fluorescence voids. (e) Zoomed in image from field in (D). (f) FRAP experiments showing high lateral mobility of DOPC in the membrane surrounding the voids. (g) AFM topograph of LPS-treated SLB showing pits of ~4 nm depth. Scale bars represent: (a) 100 µm, (b) 5 µm, (c) 1 µm, (d) 100 µm, (e) 10 µm, (f) 5 µm, (g) 10 µm.

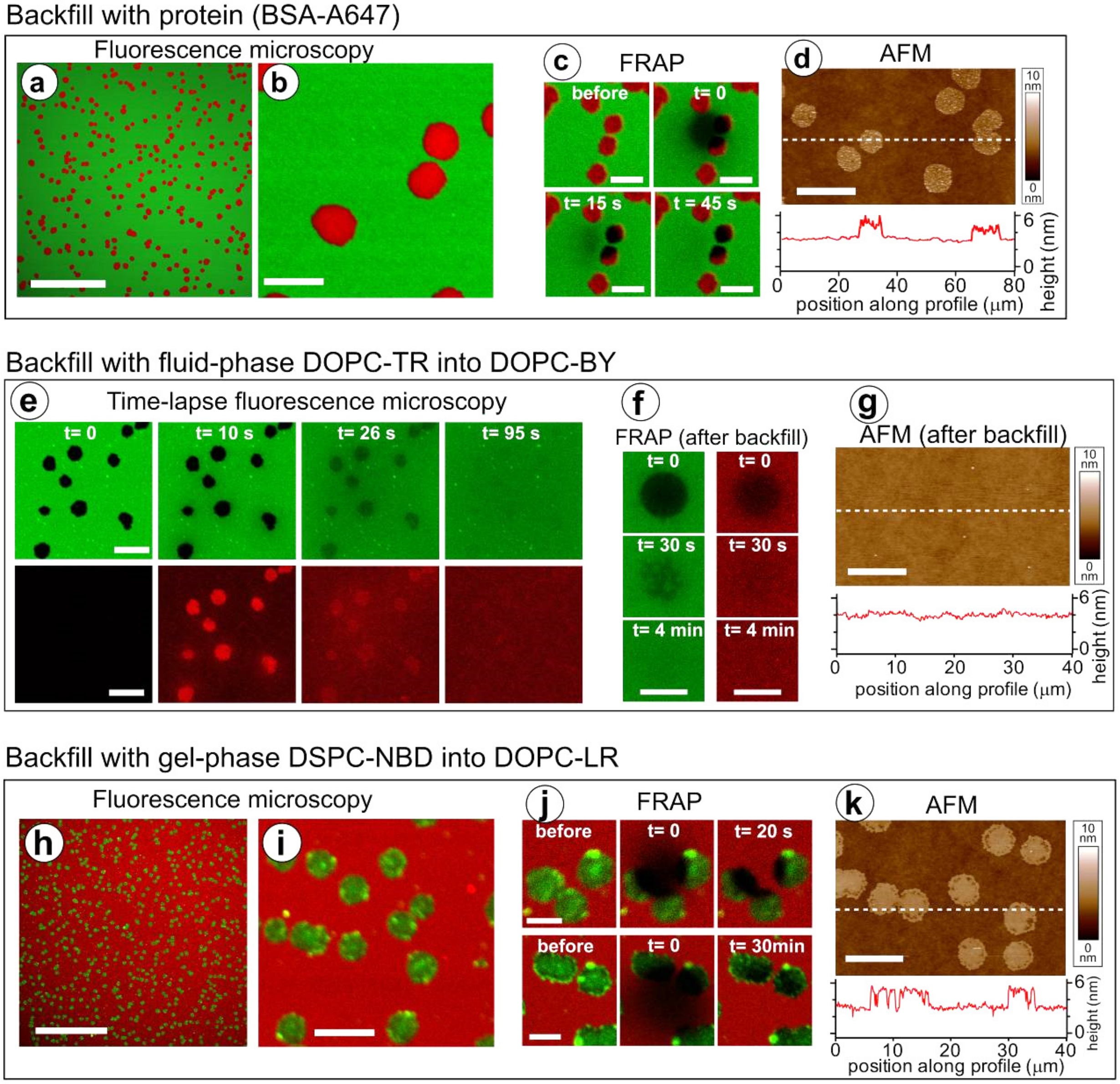

Figure S2. Multi-component membranes generated by backfilling with various secondary components. (a) LSCM showing a DOPC SLB (doped with 0.5% HPC-BY) after LPS treatment and backfilling with bovine serum albumin tagged with AlexaFluor647 (BSA-AF647). Fluorescence intensities confirm that the protein is confined to the voids (*red*) and that lipids (*green*) are excluded (b) Higher magnification image from (a). (c) FRAP experiment, with composite dual channel images, bleaching of both HPC-BY lipids and BSA-AF647 (using 488 and 633 nm excitation). Recovery of HPC-BY fluorescence in the DOPC membrane indicating high lateral lipid mobility is observed; the BSA-AF647 fluorescence does not recover indicating that the protein is static. (d) AFM and height profile (*below*) showing that the protein completely fills the voids, leading to a protruding film ~2 nm above lipid bilayer that is otherwise continuous and intact. (e) Time-lapse LSCM images showing backfilling of an LPS-treated DOPC SLB (+0.5% HPC-BY; *green*) with fluid-phase DOPC liposomes doped with a different

lipid dye (0.5% Texas Red DHPE; *red*), separate image channels shown. Incoming red lipids initially localized at the void regions (t = 10 s) followed by an outward spreading into the membrane with concomitant inwards migration of the original green lipids (t = 26 s) until the two lipid dyes are homogenously mixed (t = 95 s). (f) FRAP of a region from (e), complete recovery is observed of fluorescence for both dyes after bleaching an area that previously spanned multiple voids, suggesting that the SLB regained its continuity and fluidity after backfilling with fluid-phase lipids. (g) Representative AFM topograph of sample from (E), showing a flat surface without voids, confirming that the membrane had been repaired. (h) LSCM showing a DOPC SLB (+1% DOPE-LR; *red*) after LPS treatment and backfilling with DSPC liposomes (+1% DSPE-NBD; *green*). Fluorescence intensities confirm that the DSPC forms stable domains. (i) Higher magnification image from (h). (j) FRAP experiment, with composite dual channel images, bleaching of both DOPE-LR and DSPE-NBD (using 543 and 488 nm laser lines). Recovery of DOPE-LR fluorescence in the DOPC membrane indicating high lateral mobility is observed; in contrast the DSPE-NBD fluorescence recovers very slowly (limited recovery after 30 min) indicating that the DSPC is in the gel-state. (k) AFM and height profile (*below*) showing protruding DSPC domains ~2 nm above the DOPC lipid bilayer. Scale bars represent: (a) 100 µm, (b) 10 µm, (c) 5 µm, (d) 20 µm, (e) 10 µm, (f) 20 µm, (g) 10 µm, (h) 100 µm, (i) 10 µm, (j) 5 µm, (k) 10 µm.

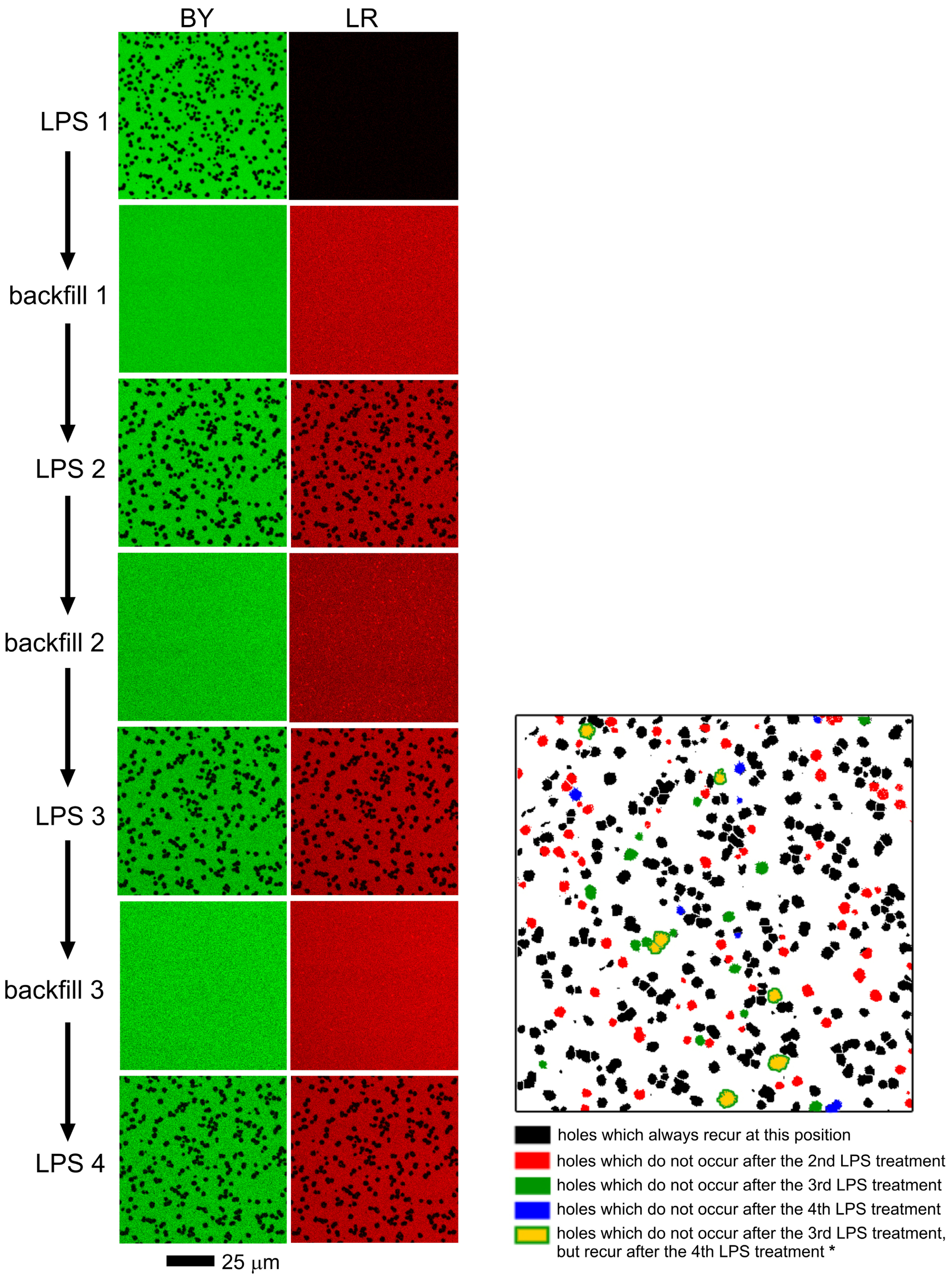

Image	Total #holes	Recurring #holes	Absent' #holes	Re-recurring #holes*	Recall ratio (overall)
LPS 1	366	-	-	-	-
LPS 2	281	281	85	0	0.76
LPS 3	261	261	20	0	0.71
LPS 4	258	251	10	7	0.70

Figure S3. Memory of LPS-induced void pattern after backfilling with DOPC. The original DOPC SLB was doped with 0.5% HPC-BY (*green*) and each backfill was performed with DOPC doped with 0.5% DOPE-LR (*red*). *Columns* show related images from each color channel: HPC-BY (*left*), DOPE-LR (*right*). *Rows* show data from a sequence of experiments, from top to bottom, cumulative number of LPS treatments (*LPS* #) and backfills (*backfill* #) as labelled. After each backfill an apparently clean membrane is regenerated, however, further LPS treatments reproduce holes at the same positions as previously. Inset *bottom-right*, is an image of overlaid data from the LPS 1-LPS 4 images, showing position of recurring, non-recurring and holes which 'disappear' and then later re-occur (see *), color-coded as labelled (generated using ImageJ). A table (*inset, at bottom*) shows the numerical analysis of this 'memory' effect, tallying the number of recurring holes, non-recurring holes, re-recurring (*) holes and the recall rate. The majority of voids were reproduced between backfill stages, but some voids are 'lost' and occasional reappearing voids were found. This experiment is similar to that of Figure 1D, except that we maintained a single field of view between stages in the current dataset, with the advantage of allowing us to track individual voids, but the disadvantage of bleaching. Bleaching occurred due to repeated imaging of the same field, therefore the fluorescence intensity data was not analysed for this dataset (instead, see Figure 1D) and the contrast was adjusted in each image for clarity.

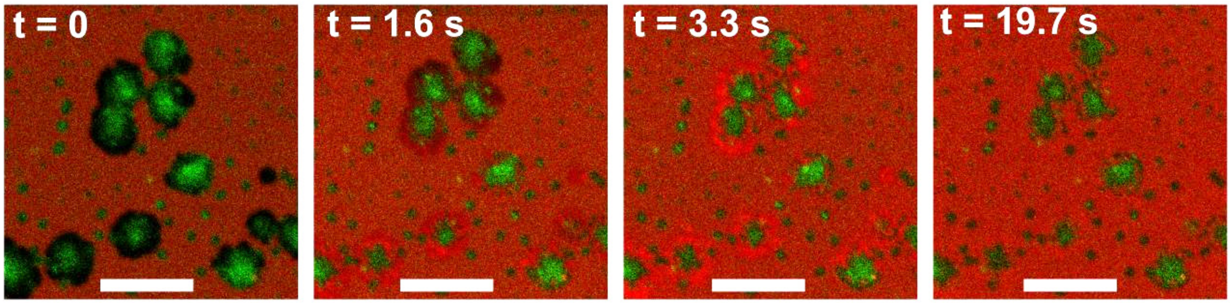

Figure S4. Multi-stage backfilling showing fluid-phase membrane regeneration. Time-lapse LSCM images showing a second backfilling with fluid-phase DOPC liposomes (*red*) into a multi-component membrane comprised of a DOPC SLB (*red*) which has already been treated as follows: (i) LPS treatment, (ii) backfilled with DSPC (*green*), (iii) second LPS treatment (DOPC doped with 1% DOPE-LR; DSPC doped with 1% DSPE-NBD). This sample (at t=0) is equivalent to that shown Figure 2e in the main text. Note that the fresh backfilled DOPC localizes in the void region at the periphery of the DSPC domain (t = 3.3 s), appearing brighter than the original DOPC-LR (due to slight bleaching of the SLB during the previous stages of the experiment) and then spreads outwards to regenerate a continuous DOPC phase. Scale bars represent 10 μm.

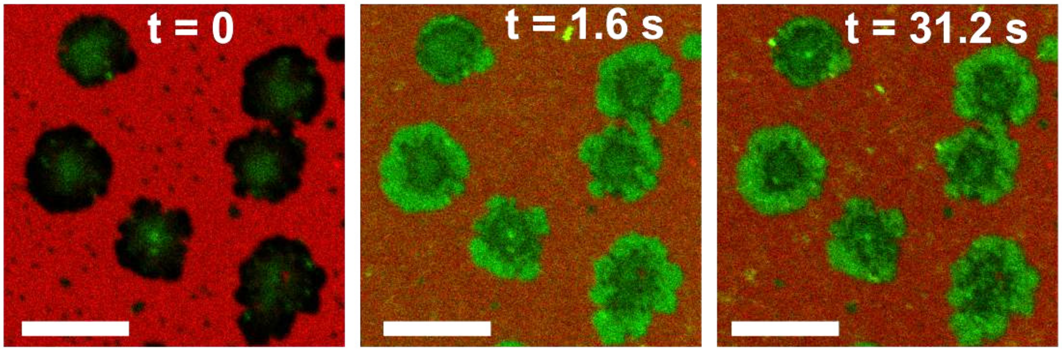

Figure S5. Multi-stage backfilling for enlarged gel-phase domain formation. Time-lapse LSCM images showing a second backfilling of a similar sample to Figure S4, except backfilling with gel-phase DSPC liposomes (*green*). Note that the fresh backfilled DSPC-NBD enlarges the DSPC domain and remains static, also appearing brighter than the original DSPC-NBD (due to significant bleaching of the SLB during the previous stages of the experiment). Scale bars represent 10 µm.

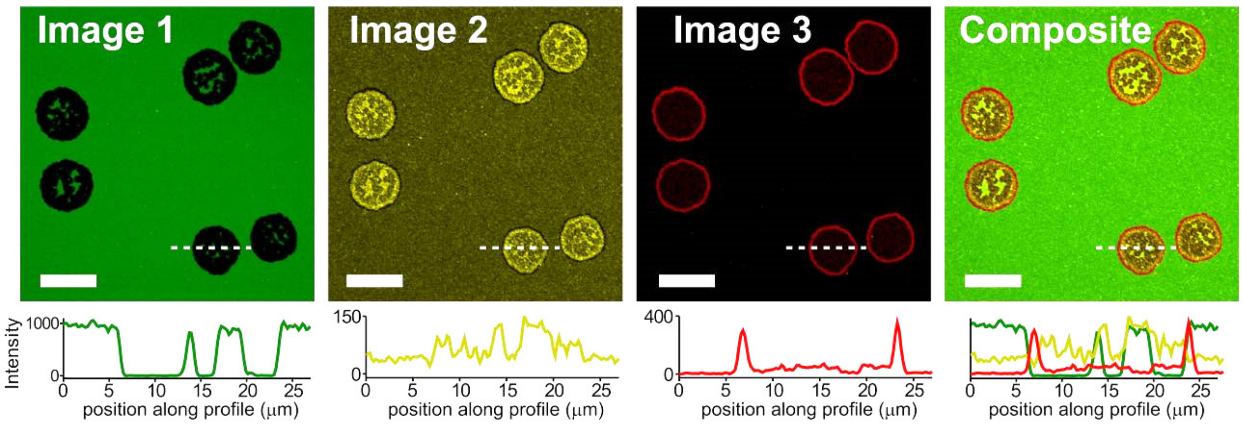

Figure S6. Multi-stage backfilling for hierarchal protein domain formation. LSCM images of a representative field of view of the arrangement of proteins and lipids after multiple cycles of backfilling with fluorescently-tagged BSA protein. This was generated by LPS treatment of a DOPC SLB (doped with 0.5% HPC-BY; *green*), followed by backfilling with BSA-AF594 (*yellow*), treating a second time with LPS, followed by a second backfilling with BSA-AF647 (*red*). Sequential images of the same area of the membrane were acquired with preferential excitation and filter sets to capture the fluorescence from a single component and to minimize overlapping signals due to spectral overlap between AF594 and AF647. *Image 1*, excitation at 488 nm, collection at 505-525 nm; *Image 2*, excitation at 543 nm, collection at 560-620 nm; *Image 3*, excitation at 633 nm, collection at 655-755 nm; *Composite*, merged images 1-3. Fluorescence intensity profiles (*below*) show counts across a section spanning a protein domain. Scale bars represent 20 μm.

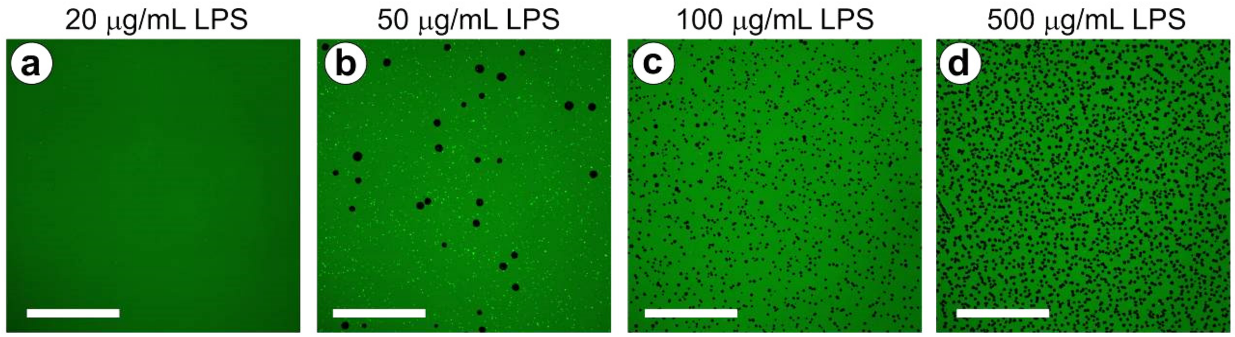

Figure S7. Concentration dependent void formation induced by LPS. LSCM images showing four DOPC SLBs (doped with 0.5% HPC-BY; *green*) after treatment with LPS at increasing concentrations, as labelled, from (a)-(d). All scale bars represent 100 μm.

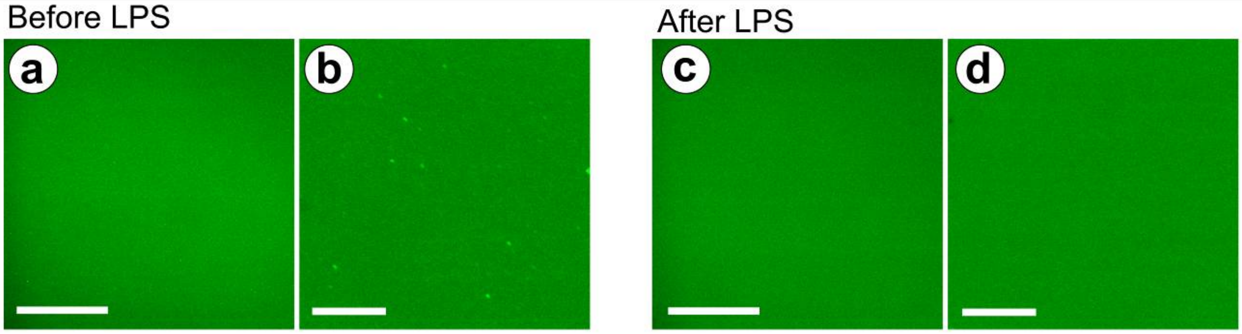

Figure S8. The resistance of DOPC lipid monolayers to LPS. A DOPC/HPC-BY lipid monolayer, formed after liposome fusion onto a silanized glass substrate, shown by LSCM at low magnification (a) and high magnification (b). The same sample after treatment with LPS, imaged at low magnification (c) and high magnification (d). There is no noticeable change to the fluorescence distribution or intensity between the images. Scale bars represent: (a), (c) 100 µm; (b), (d) 10 µm.

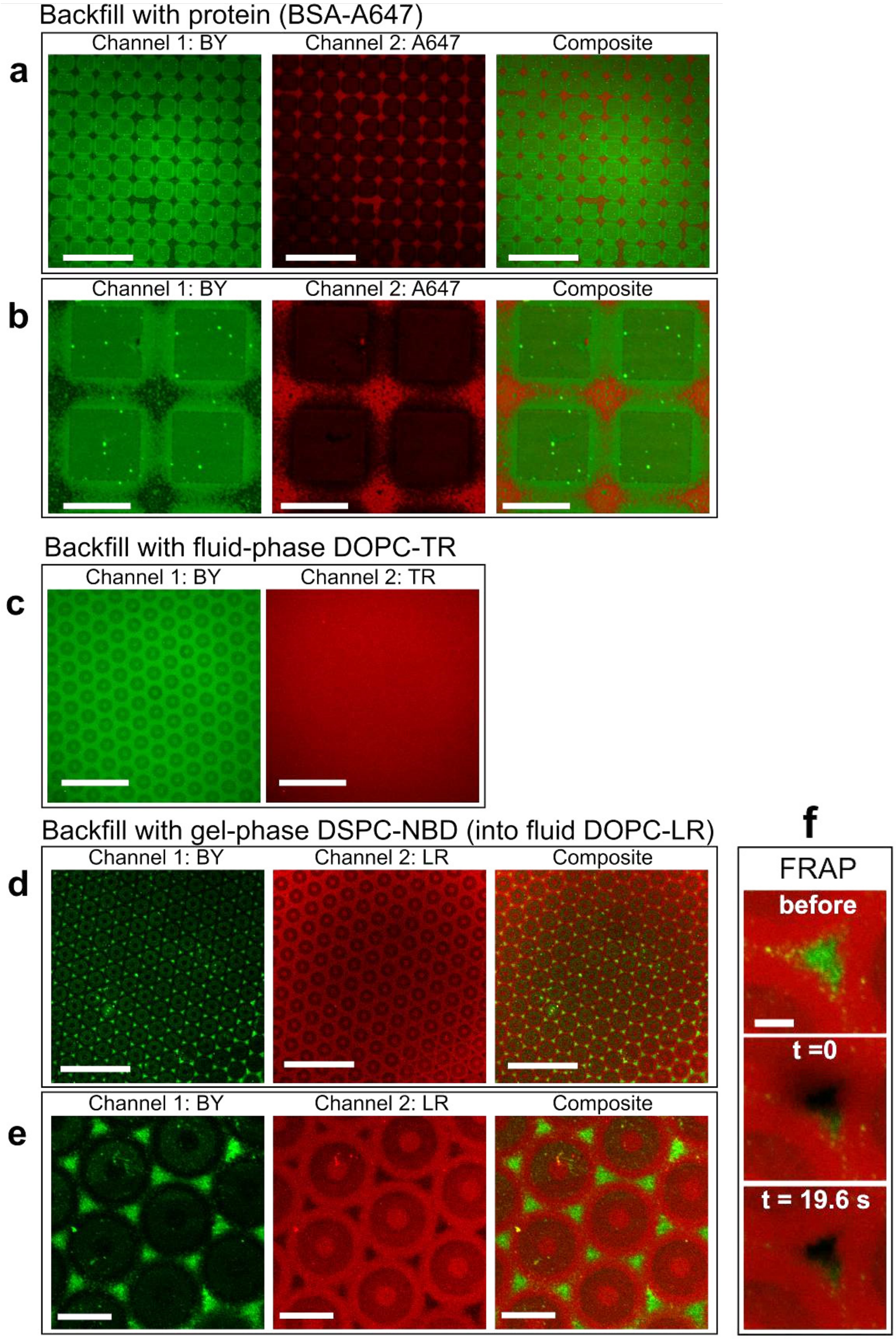
Backfill with protein (BSA-A647)
a
Channel 1: BY
Channel 2: A647
Composite
b
Channel 1: BY
Channel 2: A647
Composite
Backfill with fluid-phase DOPC-TR
c
Channel 1: BY
Channel 2: TR
Backfill with gel-phase DSPC-NBD (into fluid DOPC-LR)
d
Channel 1: BY
Channel 2: LR
Composite
e
Channel 1: BY
Channel 2: LR
Composite
f
FRAP
before
t =0
t = 19.6 s

Figure S9. Further multi-component array-patterned membranes. Dual channel LSCM data of a patterned DOPC (+0.5% HPC-BY) lipid bilayer/monolayer box array pattern (*green*) after LPS treatment and backfilling with BSA-AF647 (*red*), shown as individual channels and composite merged images, at low magnification (a) and high magnification (b). Dual channel LSCM data of a patterned DOPC (+0.5% HPC-BY) lipid bilayer/monolayer washer array pattern (*green*) after LPS treatment and backfilling with DOPC liposomes (+0.5% Texas Red DHPE; *red*), shown as individual channels (c). Dual channel LSCM data of a patterned DOPC (+1% DOPE-LR) lipid bilayer/monolayer washer array pattern (*red*) after LPS treatment and backfilling with DSPC liposomes (+1% DSPE-NBD; *green*), shown as individual channels and composite merged images, at low magnification (d) and high magnification (e). FRAP experiment, with composite dual channel images (f), bleaching of both LR and NBD (using 543 and 488 nm laser lines). Image contrast adjusted for clarity in (f). LR (DOPC) recovery is much greater than that of NBC (DSPC) after 19.6 s, indicating that the DSPC is in the gel-state. Scale bars represent: (a) 100 µm, (b) 20 µm, (c) 100 µm, (d) 100 µm, (e) 20 µm, (f) 5 µm.
